# Treatment of gingival recession with vestibular incision subperiosteal tunnel access and advanced platelet-rich fibrin

**DOI:** 10.1186/s12903-024-05398-w

**Published:** 2025-01-14

**Authors:** Marwa Abdelhaleem, Wafaa Saleh, Samah Elmeadawy

**Affiliations:** 1 Oral Medicine, Periodontology, Diagnosis and Oral Radiology Department, Faculty of Dentistry, Horus University, New Damietta, Egypt; 2 Oral Medicine, Periodontology, Diagnosis and Oral Radiology Department, Faculty of Dentistry, Mansoura University, Mansoura, 33516 Egypt

**Keywords:** Gingival recession, A-PRF, VISTA technique

## Abstract

**Objectives:**

The current literature about the effect of advanced platelet rich fibrin(A-PRF) with vestibular incision subperiosteal tunnel access (VISTA) technique in treating gingival recession is scarce. Therefore, the aim of the current randomized clinical trial is to evaluate the effect of A-PRF with VISTA technique in the treatment of Cairo class 1 gingival recession (RT1).

**Methods:**

Twenty-four patients who met the eligibility criteria were randomly allocated into two groups. VISTA + A-PRF was the treatment of the study group, while VISTA + collagen matrix was performed for the control group. The clinical outcomes were assessed by a single-blind assessor at baseline, three months, and six months. They were divided into primary and secondary outcomes. The primary outcomes included recession depth (RD), recession width (RW), gingival thickness (GT), mean of root coverage % (MRC%), and width of attached gingiva (WAG) while the secondary outcome included clinical attachment level (CAL).

**Results:**

The primary outcomes analysis demonstrated statistically significant improvements in RD, RW, MRC%, GT, and WAG after 3 and 6 months in both groups (*p* < 0.001). However, the study group demonstrated a significantly greater improvement than the control group in RD, RW, and MRC%. No significant differences were observed between the two groups regarding GT and WAG.

**Conclusions:**

Both treatment approaches were effective in the treating of RT1 adjacent gingival recessions. A-PRF showed promising results compared to collagen matrix.

**Trial registration:**

The current randomized clinical trial was registered at ClinicalTrials.gov (Registration number: NCT06357351) and it was released on 10/04/2024.

## Introduction

Migration of the gingival tissue in the apical direction results in exposure of the root surface and it is known as gingival recession. Multiple etiologic factors of gingival recession have been established including periodontal diseases, traumatic tooth brushing, mal-aligned teeth, and improper orthodontic treatment. Treatment of gingival recession involves both surgical and non-surgical approaches [1–3].

Periodontal surgical therapy of gingival recession has gained more attractiveness in recent years due to esthetic concerns. The selection of the surgical technique is mainly affected by the local anatomical considerations of the involved site [1, 4]. The Coronally advanced flap (CAF) is the most commonly used technique for coverage of the root exposure. Multiple adjustments of the CAF were applied including the combination of several grafts, and barrier membranes [5].

Among the numerous graft membranes for coverage of the gingival recession, connective tissue graft (CTG) gained popularity as the gold standard and the maximum predictable grafting material. However, it is a time-consuming technique and requires a second surgical site with restricted tissue availability. In addition, post-operative pain results in more patient discomfort [6]. Consequently, treating gingival recession demands finding a substitute soft tissue graft material such as collagen matrix and platelet concentrates [7].

The vestibular incision subperiosteal tunnel access (VISTA) technique is a minimally invasive approach that has been recently introduced for treatment of gingival recession [8]. It maintains the vascularization of the targeted area while maintaining the integrity of the marginal gingiva and preserving the architecture of the interdental papilla during the healing after periodontal surgery. In addition, it provides wide access to the surgical area through the vertical incision with minimal scaring eventually improving the esthetic outcomes [9].

Platelet concentrates are now widely used in tissue repair stimulation and cellular regeneration in several medical and dental fields [10–13]. They had been produced via centrifuging blood to split it into constituent cells, platelet concentrates enmeshed into a fibrin matrix, and materials that promote cellular division. Different classes were attained from the platelet concentrate by changing the centrifugation process with different cells and structures of the fibrin network [14].

Advanced platelet-rich fibrin (A-PRF) was obtained by reducing the centrifugation speed and increasing the centrifugation time. It is a new membrane with a homogeneous distribution of the platelets, leukocytes, and cells throughout the entire membrane. Because A-PRF has a higher concentration of growth factors and cytokines than older versions of PRF, including platelet-derived growth factor (PDGF) and insulin-like growth factor 1 (IGF-1), it directly accelerates tissue regeneration [15, 16].

While the current literature has demonstrated the benefits of the independent use of the VISTA technique and A-PRF in the field of gingival recession treatment with consideration of the above-mentioned advantages of A-PRF and limitations of CTG [17–19], our study aimed to combine the effects of both modalities to obtains benefits of VISTA technique and the regenerative potential of A-PRF as well as an effective and patient-friendly approach. This study aims to systematically compare the augmented effect of A-PRF and VISTA technique to the collagen matrix in conjunction with the VISTA technique for the treatment of gingival recessions (Cairo class I).

## Materials and methods

### Patients’ selection

The participants of the current randomized clinical trial were chosen from patients seeking coverage of the exposed root surfaces and attending the Periodontology clinic at the Department of Oral Medicine and Periodontology at the Faculty of Dentistry, Mansoura University, Egypt. The participants were initially assessed for inclusion in our study according to the predesigned eligibility criteria.

The current study was approved by the human subject ethical board at the Faculty of Dentistry, Mansoura University (Approval number: "A29080622″) and conducted in accordance with the Helsinki Declaration of 1975, as revised in 2013.”

The clinical procedures were explained to the participants including the potential complications. Then, the informed consents were obtained from them before starting the procedures of the study. The current randomized clinical trial was registered at ClinicalTrials.gov (Registration number: NCT06357351).

### Inclusion and exclusion criteria

We included cases with single and multiple adjacent gingival recession type (RT1) Cairo Classification in which the interproximal CEJ is not clinically detectable. The participants who were systemically healthy with good oral hygiene and aged from 18–50 years were included in the current study. The patient’s age was selected based on the aim of obtaining homogenous healing among all patients. Patients below 18 years of age have immature periodontal tissues while patients older than 50 years of age have several factors that may impact the periodontal healing after surgery including systemic diseases, slow cellular turnover, and compromised healing [20–24].

Good oral hygiene patients were selected based totally on the subsequent standards: (1) Plaque Index score of < 1.0, indicating low degrees of plaque accumulation (Silness and Löe, 1964); (2) Gingival Index rating of < 1.0, reflecting slight or no gingival inflammation (Löe and Silness, 1963); and bleeding on Probing in < 10% of sites showed ginigval bleeding, following Ainamo and Bay’s classification for minimal gingival bleeding" [25–27].

The following exclusion criteria were applied in our study including patients with systemic diseases interrupting the healing after surgical procedures, patients who showed improper oral hygiene, smoking, and alcohol consumption, patients with allergy to the biomaterials used in the current study, patients with cervical restoration in the facial surface of the involved tooth, patients with high frenal pull and patients received a periodontal surgery at the same location of designed treatment of gingival recession in the in the past 1 year.

### Sample size calculation

The current study was conducted in referring to a previous study that evaluated the effect of A-PRF in gingival recession treatment, in which the increase in the gingival recession height was evaluated at 3 and 6 months postoperatively [9]. We included two groups; the study group received VISTA technique with A-PRF, and the control group received VISTA technique and collagen matrix. We used the G Power program version 3.1.9.4 with the expected effect size of 1.42(magnitude of the expected difference between the two groups of our study) based on the prior research [9] and a 2-tailed test with an α error set at 0.05 and a power of 80.0%. The findings of the sample size calculation showed that 12 patients were required in each group.

### Random distribution and blinding process

The participants of the current study were randomly allocated to either the study or the control groups using a computer-generated sequence. They were allocated to study group (VISTA + A-PRF) or control group (VISTA + Collagen matrix). Each number of participants was placed in an opaque sealed envelope which was only opened by the surgeon immediately before the surgery while treatment for each participant was disclosed only at the time of surgery. One surgeon performed all surgeries to maintain the clinical setting and standardize the surgical procedures.

### The study outcomes

A single-blinded assessor measured the study outcomes at baseline, 3 months, and 6 months after the surgical treatment. The outcomes were divided into primary and secondary outcomes. The primary outcomes included Recession depth(RD), recession width (RW), width of keratinized gingiva (WKG), gingival thickness (GT), and mean root coverage% (MRC%) while the secondary outcome included clinical Attachment Level (CAL).

The following primary outcomes were measured as follows: RD was measured as the distance from the lowest point of free gingival margin to CEJ and RW was assessed at the level of CEJ. The WKG was measured as the distance from the mucogingival junction to the outer surface of the gingival sulcus. GT was assessed at an exact point on the treated tooth using K-endodontic file #15 under local anesthesia. The file was placed perpendicularly 3 mm below the gingival margin until the felling of hard tissue. The silicon disc stopper was secured, and the file was measured with digital caliber.

MRC% was calculated using the following formula

$$\text{Mean root coverage formula}\; \%=\frac{\text{Pre opeative recession depth}-\text{Post operative recession depth}}{\text{pre operative recession depth}}\times100$$

A UNC-15 probe was used to assess CAL from the CEJ to the pocket's base. The primary and secondary outcomes were recorded at baseline, 3 months, and 6 months after surgery.

The primary and secondary outcomes of the current study were recorded by the same outcome assessor which was blinded to the study groups, or the treatment procedures performed for each participant to avoid an unbiased evaluation of the study's outcomes. Intraexaminer calibration was assessed on two separate occasions with 48-h intervals prior to the real measurement.

### Surgical procedure for gingival recession treatment

All the participants received comprehensive phase 1 therapy including thorough scaling and root planning, oral hygiene instructions and reinforcement of plaque control one month before surgery. The surgical site was irrigated by topical povidone-iodine 0.5% (Betadine, El-Nasr Pharmaceutical Chemicals Co., Egypt) and received local anesthesia of 1.8 ml using 4% articaine with 1:100.000 epinephrine (Artinibsa®, Inibsa Dental, Egypt). We started the VISTA technique with an 8 to 10-mm vertical incision extending from the mobile mucosa and reaching the apical end of the keratinized gingiva. A scalpel was used to make intrasulcular incisions covering up to a third of the papilla’s width medially and distally. The subperiosteal tunnel flap was elevated using a small subperiosteal elevator that was inserted through the vertical incision. The tissue of mobile and immobile mucosa around the affected tooth was included in the flap extending at least one tooth beyond the teeth which required root coverage. These procedures were performed utilizing the VISTA kit (Devmed tunnel instrumentation).

After elevation of the flap, the A-PRF membrane and the collagen matrix (Bio-resorbable bovine non-cross-linked collagen matrix (TUTOPATCH tissue matrix –Tutogen Medical GmbH, Germany) were placed through the vertical incision without additional fixation.

This approach was selected to decrease manipulation of the membranes, minimize trauma of the tissues, maintaining the vascular integrity of the tissues, and enhance healing after surgery. In addition, we depended on the design of the VISTA technique to maintain the collagen matrix and A-PRF in place. It provides a stable and well vascularized space for the utilized biomaterial without the need for an additional fixation [9, 28, 29].

**Fig. 1:**
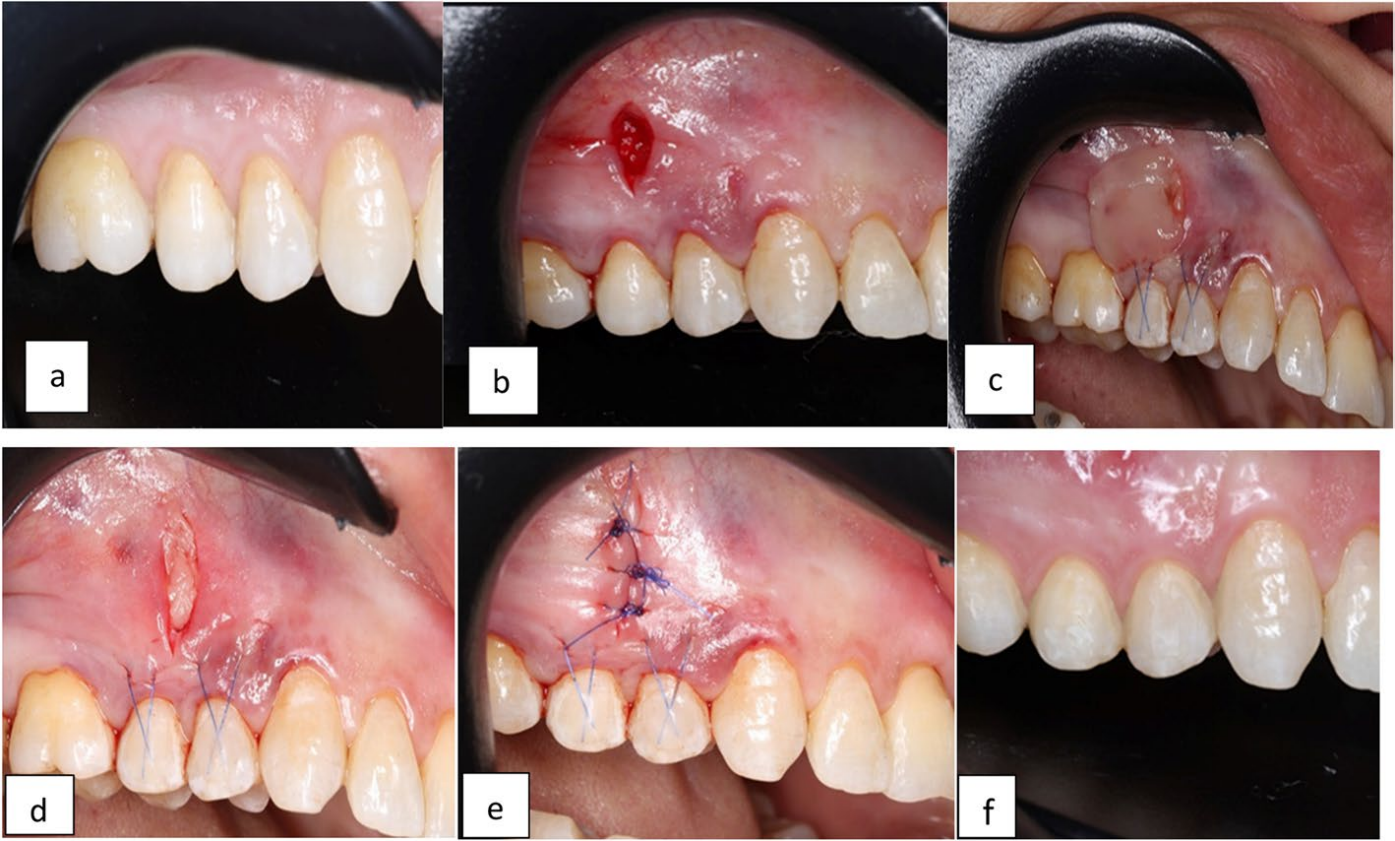
Shows a case presentation of Group (I). **a** Preoperative view of the gingival recession in upper premolars. **b** Vertical incision extending to the MGJ. **c **Placement of A-PRF membrane. **d** Shows the A-PRF on site. **e** Shows suturing of the vertical incision. **f** Shows the treated site after a 6-month follow-up period

**Fig. 2:**
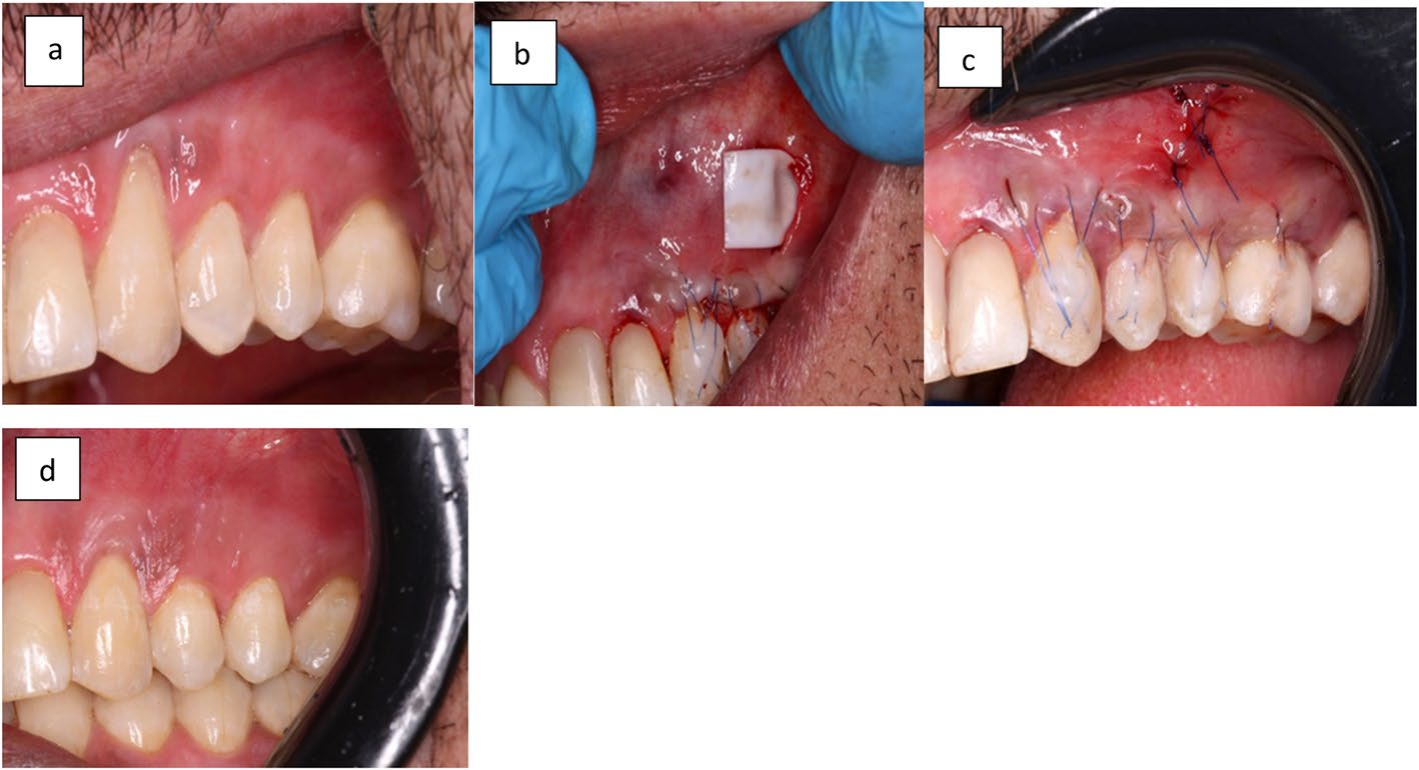
Shows a case presentation of Group (II). **a** Preoperative view of the gingival recession in upper in the upper left first and second premolars. **b **Placement of collagen matrix. **c** Shows suturing of the vertical incision. **d** Shows the treated site after a 6-month follow-up period

Each tooth was prepared for suture attachment, using a brief EDTA gel (PrefGel® (Straumann®, Basel, Switzerland)) for etching of the facial enamel surface followed by thorough rinsing and drying. To prevent the gingival margin from moving apically during initial healing, 5.0 monofilament polypropylene sutures (Surgipro® by Medtronic (Medtronic, USA).) were fixed to the facial aspect of each tooth with a small amount of flowable composite resin over the knot. The vertical incision was then closed and sutured using multiple 5.0 monofilament polypropylene sutures, and a periodontal dressing Coe-Pak (GC Dental, Egypt) was applied. Figs. 1 & Fig 2.

### Preparation of the A-PRF

Twenty milliliters of antecubital vein blood was drawn intravenously. The withdrawn blood was centrifuged for 14 min at 1500 rpm using IntraSpin® system by Intra-Lock International, Inc. After centrifugation, three layers were created while the middle layer is the A-PRF membrane. The A-PRF clot layer was gently compressed to form the A-PRF membrane then the membrane was placed within the subperiosteal tunnel through the vertical incision and extended both mesially and distally.

### Postoperative care instructions

After surgery, All the patients were prescribed analgesics twice daily (Diclofenac Potassium 50 mg tablets). Patients were instructed to maintain optimal oral hygiene with Chlorhexidine digluconate mouth rinse (0.12%) and received oral hygiene instructions.

Brushing the surgical site was stopped for four weeks while the patients were instructed to rinse their mouths with 0.2%chlorhexidine gluconate mouthwash daily for two weeks. Two weeks after surgery, the sutures and the residual composite were removed.

### Statistical assessment

Data were analyzed by IBM SPSS software package version 20.0. (Armonk, NY: IBM Corp). Qualitative data were described using numbers and percentages. The Shapiro–Wilk test was used to verify the normality of distribution. For the comparison of categorical variables of different groups, the Chi-square test was used. For normally distributed quantitative variables, ANOVA was used to compare between more than two periods (baseline, 3 months, and 6 months). For pairwise comparisons following the ANOVA, a Bonferroni post-hoc test was applied using the second approach of the Bonferroni correction. In addition, the student t-test was used for normally distributed quantitative variables, to compare between two studied groups.

## Results

**Fig. 3:**
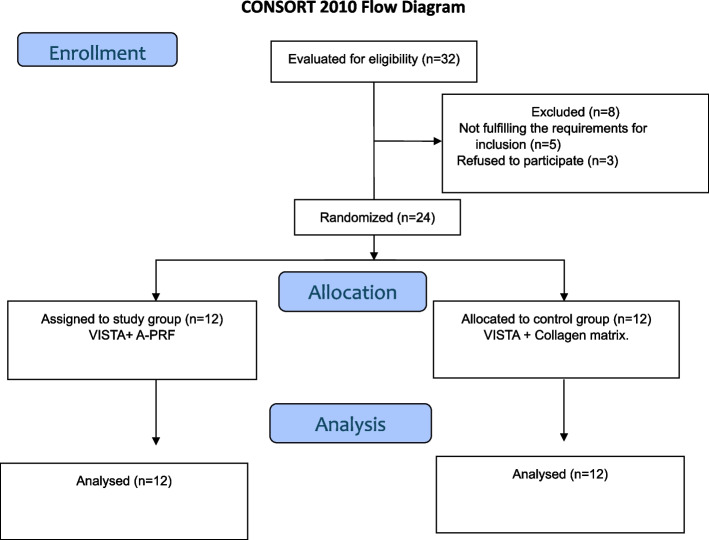
CONSORT flow chart for trial recruitment

After an initial assessment of the patients, 32 patients were first included. Then 8 patients were omitted of which 5 participants didn’t qualify according to the eligibility criteria while 3 participants refused to participate after the conversation about the study procedures. Figure 3 Twenty-four patients with an age range from 21 to 47 years old were included. The current study was applied on 46 teeth (anterior teeth and premolars) with single or multiple gingival recessions. The operated teeth were 36 maxillary teeth and 10 mandibular teeth. We found no significant differences in the patient's age, gender, or number, and location of treated teeth with single or multiple gingival recessions among the study groups (Table 1).

**Table 1:** Demographic comparison of two studied groups

	**(VISTA + A-PRF)** **(*n* = 12)**		**(VISTA + collagen membrane)** **(*n* = 12)**		**Test of Significance**	**p**
	**No**	**%**	**No**	**%**		
**Gender**					χ^2^ = 1.510	0.219
Male	7	58.3	4	33.3		
Female	5	41.7	8	66.7		
**Age (years)** Mean ± SD	42.0 ± 5.48		40.33 ± 5.25		t = 0.761	0.455
**Number of treated teeth**	**Single recession:3**		**Single recession:5**		χ^2^ = 0.438	0.508
	**Multiple recession:22**		**Multiple recession:16**			
**Location of treated teeth (anterior and premolars)**	**Maxillary:21** **Mandibular:4**		**Maxillary:15** **Mandibular:6**		χ^2^ = 0.912	0.340

t: Student t-test

χ^2^: Chi-square test

Table 2 was utilized to compare the RD and RW between the two groups over the entire course of the investigation. Regarding the intergroup comparison of RD and RW, there was a statistically significant difference in mean RD and RW between both groups at 3 and 6 months with *P* value < 0.001. At the baseline, there was not a significant difference between both groups. However, in the intragroup comparison of the baseline and three-month values in group I and group II, RD and RW differed significantly with a *P*-value (*p* < 0.001). In addition, there was a significant difference between baseline and 6 months as the mean of RD and RW with a *P* value of (*P* < 0.001). However, there is no major difference between 3 and 6 months (Table 2).

**Table 2:** Comparison of the primary outcomes among the study groups

Parameter	Periods	(VISTA + A-PRF) (*n* = 12)	(VISTA + collagen matrix) (*n* = 12)	Intergroup comparison of *P*-value at the same point of time	Intragroup comparison of *p*-value	
					**Group (I)**	**Group (II)**
**Recession Depth**	Baseline	2.73 ± 0.52	2.81 ± 0.40	0.682	p1 < 0.001^*^	p1 = 0.001^*^
	3 months	0.76 ± 0.13	1.19 ± 0.37	< 0.001^*^	p2 < 0.001^*^	p2 < 0.001^*^
	6 months	0.88 ± 0.20	1.38 ± 0.29	< 0.001^*^	p3 = 0.142	p3 = 0.243
**Recession Width**	Baseline	4.44 ± 0.71	4.42 ± 0.85	0.959	p1 < 0.001^*^,	p1 = 0.027^*^
	3 months	2.16 ± 0.52	3.55 ± 0.41	< 0.001^*^	p2 < 0.001^*^,	p2 = 0.018^*^
	6 months	2.30 ± 0.44	3.85 ± 0.47	< 0.001^*^	p3 = 0.123	p3 = 0.064
**Width of Attached gingiva**	Baseline	4.17 ± 0.68	4.65 ± 1.02	0.194	p1 < 0.001^*^,	p1 < 0.001^*^
	3 months	5.31 ± 0.49	5.57 ± 0.92	0.396	p2 < 0.001^*^,	p2 < 0.001^*^
	6 months	5.17 ± 0.70	5.20 ± 0.95	0.910	p3 = 0.317	p3 = 0.149
**Gingival thickness**	Baseline	1.48 ± 0.32	1.52 ± 0.37	0.111	p1 < 0.001^*^	p1 < 0.014^*^
	3 months	1.92 ± 0.41	1.78 ± 0.25	0.087	p2 < 0.001^*^	p2 < 0.018^*^
	6 months	1.99 ± 0.34	1.77 ± 0.23	0.223	p3 = 0.971	p3 = 0.687
**Mean of Root coverage%**	3 months	71.07 ± 6.92	56.59 ± 15.35	< 0.001^*^		
	6 months	67.26 ± 8.19	50.09 ± 11.42	< 0.001^*^		

p: *p* value for comparing between both groups

p1: is the comparison of *P*-value between Baseline and 3-Months of the same group

p2: is the comparison of *P*-value between Baseline and 6-months of the same group

p3: is the comparison of *P*-value between 3-Months and 6-Months of the same group

^*^Statistically significant at *p* ≤ 0.05

Despite the WAG and GT among the two groups showing no statistically considerable differences between both groups at baseline, 3 months, and 6 months, the mean difference in WAG and GT in group (I) was greater than in group (II) at 3 months and after 6 months. In the intragroup comparison, both group (I) and group (II) showed statistically significant differences between baseline and 3 months, baseline and 6 months (*p* < 0.001). In addition, we found significant differences in MRC% between both groups at the same point of follow-up (3 months and 6 months) however, both groups showed no intragroup difference through the follow-up periods (Table 2).

The secondary outcome examined in this study included only CAL. The comparison of CAL among the study groups showed a statistically insignificant difference in CAL between both groups at baseline but after 3 months there was a noteworthy reduction in CAL between groups (I)&(II) in favor of group (I) (*P* < 0.001). Then after 6 months, there was an increase in CAL in both groups but was elevated in group (II) than in group (I) with a statistically significant variation between both groups (*P* < 0.001). Moreover, the intragroup comparison between groups (I) & (II) in 3-time intervals of the study showed that in group (I) there was a notable statistical reduction in CAL between baseline and 3 months (Table 3).

**Table 3:** Comparison of the secondary outcome among the study groups

Parameter	Periods	(VISTA + A-PRF) (*n* = 12)	(VISTA + collagn matrix) (*n* = 12)	Intergroup comparison of *P*-value at the same point of time	Intragroup comparison of *p*-value	
					**Group (I)**	**Group (II)**
**Clinical Attachment level (CAL)**	Baseline	4.01 ± 0.70	4.25 ± 0.78	0.430	p1 < 0.001^*^	≥ 0.05
	3 months	1.90 ± 0.56	3.43 ± 0.80	< 0.001^*^	p2 < 0.001^*^	
	6 months	2.10 ± 0.58	3.70 ± 0.80	< 0.001^*^	p3 = 0.281	

p: *p* value for comparing between both groups

p1: is the comparison of *P*-value between Baseline and 3-Months

p2: is the comparison of *P*-value between Baseline and 6-months

p3 is the comparison of *P*-value between 3-Months and 6-Months

^*^Statistically significant at *p* ≤ 0.05

## Discussion

The main purpose of gingival recession treatment is to obtain complete coverage of the exposed root while improving the gingival thickness to enhance the patient's esthetics and self-confidence. Throughout the past decades, several surgical approaches have been developed to obtain superior results of root coverage. Despite the efficacy of these techniques, various complications were reported including tissue morbidity of the donor site and less patient satisfaction [4, 30–33].

While CTG is the gold standard graft material for coverage of gingival recession, it has been associated with several limitations including the donor site morbidity, post operative pain, long healing period, and patient discomfort. All these factors make our patients hesitant to undergo an invasive surgical procedure like harvesting CTG from the hard palpate [6]. In our control group, we aimed to overcome those limitations of CTG while improving our patients’ comfort and satisfaction by providing an accessible and acceptable treatment modality to a wide range of patients.

In addition, the recent research has shown that CM is an effective alternative graft material for coverage of gingival recession defects [34–36]. It has improved the clinical and patients reported outcomes as root coverage and patient satisfaction. It acts as a three-dimensional framework while it enables the infiltration and recolonization of fibroblasts, blood vessels, and epithelium from neighboring tissues, ultimately transitioning into gingival tissue. CM easily integrates with the surrounding tissues improving the long-term esthetic and functional outcomes. Furthermore, it eliminates the need for a second surgical site while decreasing the surgical time and patient discomfort [37, 38].

With the introduction of the tunneling technique and the novel subtypes of PRF, the present study aimed to evaluate gingival recession treatment utilizing the VISTA technique with A-PRF and collagen matrix. After conducting the surgical procedure, we followed up with our patients for 6 months to measure the effect of the selected technique and biomaterials on the coverage of gingival recession.

The result of this research showed a statistically significant reduction in RD, RW and improvement of MRC% in both groups at 3- and 6-month follow-up in comparison to baseline. However, comparison of the same outcomes from 3 to 6 months of follow-up, both groups showed a slight increase in RD, RW, and a decrease in MRC which weren’t clinically and statistically significant.

The enhancement of the MRC% and reduction of RD and RW in both the study and control groups may be attributed to the surgical procedures of VISTA technique which is a conservative approach that requires the comprehensive dissection of the soft tissues and elimination of the tension of periodontal tissues. In addition, it perseveres the integrity of the interdental papilla and maintains the blood supply of the tissues. Moreover, the coronal advance of the gingiva stopped the apical movement of the gingival tissue during the healing process which is a specific criteria of VISTA techniques that makes it differ from any other tunneling techniques [39, 40].

The results of this study were comparable with other studies using the VISTA technique with different grafting materials such as the study conducted by Jain et al.2021 [9] comparing the A-PRF and collagen matrix using the VISTA technique; the MRC% after 6 months in A-PRF group was (77.50 ± 46.78) % and it was (61.67 ± 25.20) % in collagen matrix group. Another study was conducted by Durgapal and Shetty 2023 using the VISTA technique in combination with A-PRF compared to the use of VISTA and collagen matrix, which demonstrated a reduction in RD similar to the outcome of this result [28]. Chenchev et al. 2016 also performed a study on multiple adjacent gingival recessions using VISTA and PRF and this study showed comparable results to our study [41]. Hegde et al.2021 conducted another study comparing VISTA and PRF and VISTA and CTG, the RD of this study showed a significant reduction in both groups after 6 months [42].

The percentage of MRC was initially improved then decreased from 3rd month to 6th month after surgery. The reduction of MRC% in both groups may be due to the tissue remodeling phase during the late stage of periodontal healing, especially in the areas of high mechanical stress.

There was no creeping attachment among our patients. This may be due to the gingival biotype of the patients, the surgical technique used (VISTA A-PRF or collagen matrix), or the short follow-up period of 6 months. Creeping attachment is normally related to longer follow-up durations and unique grafting techniques as subepithelial CTGs, which were not used in this study [43].

The higher reduction in RD and RW in A-PRF group than collagen matrix may be attributed to the biologic properties of A-PRF which is a completely biologically compatible membrane that integrates with the surrounding periodontal tissues. It enhances tissue regeneration by the massive release of growth factors and cytokines while enhancing collagen formation as well as endorsing angiogenesis [44, 45]. A-PRF was documented to release the highest levels of growth factors which explains its ability to induce tissue regeneration and revascularization [45].

Regarding the GT, it showed improvement in groups (I) &(II). Both groups showed statistically significant GT increases at 3 months, which were maintained at 6 months. Nevertheless, the A-PRF group exhibited a more considerable increase in GT compared to the collagen matrix group. The increased thickness of the gingiva in the A-PRF group may be due to the nature of the A-PRF membrane which is a three-dimensional fibrin network in which the endothelial cells can proliferate. In addition, A-PRF releases a higher amount of growth factors like insulin-like growth factors and endothelial growth factors which stimulate the proliferation of fibroblasts and acceleration of angiogenesis and collagen synthesis. These result in a more vascular and thicker granulation tissue [46]. However, in group 2, the notable increased GT in the collagen matrix group may related to that the collagen matrix acts as a three-dimensional framework, it enables the infiltration and recolonization of fibroblasts, blood vessels, and epithelium from neighboring tissues, ultimately transitioning into gingival tissue [37, 38].

In our study, we found that both treatments effectively reduced the CAL while a more substantial reduction was detected in the A-PRF group. The improvement in CAL was a predictable outcome of recession coverage resulting from the movement of the attachment apparatus towards the coronal direction following VISTA procedures. Previous research suggests that the VISTA technique promotes the coronal migration of the gingival margin. The mechanism of the coronal coverage after VISTA technique is uncertain but may be due to the contractility of the fibroblasts and the active healing processes that enhance the coronal movement of the attachment apparatus [47, 48]. Additionally, the concentrated growth factors in the A-PRF stimulate tissue regeneration, angiogenesis, and tissue migration [49].

### Limitations

We report some limitations of the current study including the small sample size of the included patients which decreases the generalizability of the study’s outcomes. In addition, we followed-up our cases for 6 months which aimed to evaluate the short-term improvements of the gingival recession treatment outcomes. However, further studies including a larger number of patients with longer follow-up duration are recommended to generalize the applicability of both treatment modalities of gingival recession.

## Conclusion

Within limitation, our study showed that both treatment groups were efficient in improving the clinical parameters from baseline to the 3 and 6-month follow-ups. However, when compared to the combination of VISTA and collagen matrix, the use of VISTA in combination with A-PRF demonstrated more prominent improvements in clinical parameters, such as decreases in RD, RW and MRC%, and CAL. Conversely, there were no significant differences found in the GT or WAG when comparing both groups.

## Data Availability

The data that support the findings of this study are available from the corresponding author upon reasonable request.
